# Uncertainty Quantification for Cardiac Diffusion Tensor Imaging Without Additional Datasets

**DOI:** 10.1002/mrm.70414

**Published:** 2026-05-10

**Authors:** Sam Coveney, Irvin Teh, May Lwin, Mehak Asad, Isaac Watson, Maryam Afzali, Erica Dall'Armellina, Jürgen E. Schneider

**Affiliations:** ^1^ Biomedical Imaging Science Department, Leeds Institute of Cardiovascular and Metabolic Medicine University of Leeds Leeds UK; ^2^ Division of Cardiovascular Sciences, College of Life Sciences University of Leicester Leicester UK

**Keywords:** bootstrap, cardiac diffusion tensor imaging, magnetic resonance imaging, sampling distribution, uncertainty quantification

## Abstract

**Purpose:**

Cardiac diffusion tensor imaging (cDTI) is subject to physiological noise, thermal noise, and signal corruption, which cause errors in diffusion measures. While a larger dataset can be decimated to investigate the general precision of measures from fitting smaller datasets, uncertainty quantification (UQ) methods for fitting entire particular datasets are required for UQ to be output from cDTI post‐processing pipelines.

**Theory and Methods:**

To account for non‐idealized errors in cDTI, repetition bootstrap methods with whole‐image resampling are required to approximate the sampling distribution of measures. We demonstrate UQ of voxel‐wise diffusion measures and myocardial summary statistics over multiple voxels, as well as uncertainty‐weighted summary statistics and their uncertainties. Methods are demonstrated on datasets of healthy volunteers and hypertrophic cardiomyopathy patients.

**Results:**

Group differences are larger (and *p* values smaller) for MD, FA and ∣E2A∣ when myocardial averages of diffusion measures are weighted by uncertainty. This is particularly true for ∣E2A∣ (difference of group medians: $24.0^{{}^{\circ}}$ for unweighted average, $36.7^{{}^{\circ}}$ for uncertainty weighted average). The uncertainty of averages over myocardial voxels is useful to understand outlier cases where it is difficult to determine if the result is trustworthy from diffusion measures alone. Uncertainty maps are also useful for highlighting regions of less trustworthy diffusion measures.

**Conclusion:**

Uncertainty quantification in cardiac diffusion tensor imaging can be performed with respect to the sampling distribution of the available cDTI dataset, provided the dataset design is suitable for repetition bootstrapping.

**Abbreviations**
cDTI: cardiac diffusion tensor imaging
UQ: uncertainty quantification

## 1 Introduction

Cardiac diffusion tensor imaging (cDTI) is a method for characterizing myocardial tissue in vivo [1]. The design of the scan, such as the number of diffusion encoding directions and the number of repeated acquisitions, has been investigated in previous studies [2, 3, 4], most recently in [5], in order to understand the effects of scan design on the accuracy and precision of diffusion measures. While the general precision of measures for a particular acquisition design can be obtained by decimating a larger dataset [6], the current paper focuses on a different task: how to perform uncertainty quantification (UQ) of diffusion measures derived from fitting the entire available dataset. Methods that achieve this goal allow UQ to be included as an additional output for CDTI post‐processing pipelines.

In addition to obtaining uncertainty maps of diffusion measures, UQ in cDTI is also applicable to summary statistics over myocardial voxels (e.g., the variance of the mean of MD over all voxels in a slice; the variance of the median of FA over all voxels in all slices; the variance of MD in a specific region of interest such as the septum or region of myocardial infarction; etc.), since these quantities are important outputs of a cDTI post‐processing pipeline. Furthermore, since summary statistics are involved in cDTI, and the uncertainty of fitted diffusion measures varies on a voxel‐to‐voxel basis, it is possible to calculate uncertainty weighted summary statistics over myocardial voxels, which has not been considered thus far in cDTI. Here, we show how to perform UQ for diffusion measures obtained from fitting the entire available dataset, and demonstrate potential advantages on previously analyzed datasets of healthy volunteers (HV) and hypertrophic cardiomyopathy (HCM) patients.

## 2 Theory

A dataset obtained from a cDTI scan should be considered a random sample from a data generating process. Any such sample is subject to aleatoric uncertainty arising from randomness (e.g., thermal noise) and so it follows that diffusion measures calculated from different samples will vary. The probability distribution of a diffusion measure (obtained by fitting a model/signal representation to data) with respect to random samples is its “sampling distribution”. Estimating the sampling distribution is a form of uncertainty quantification: inference about the population (all potential datasets) must be made from a sample (single available dataset). This can be done with bootstrapping methods: the sample dataset can be repeatedly resampled in order to assess the variability of measures arising from the data generating process using these resampled data. Estimates of the sampling distribution are therefore comparable with reproducibility experiments which actually obtain two or more samples (datasets) from the data generating process, although repeat scans may help to assess variability from additional sources if the subject leaves and re‐enters the scanner (e.g., patient compliance, imaging plane placement, etc.). Reproducibility experiments require repeating the entire acquisition, and it is important to consider that inter‐scan variability is conflated with the general variability of the data generating process within each individual scan, that is, intra‐scan variability. Furthermore, assuming the ability to combine the repeated acquisitions together (which may not be sensible if the subject left and re‐entered the scanner), the question of the uncertainty of measures obtained by fitting all available data is left unanswered.

The sampling distribution can be contrasted with the Bayesian posterior distribution of diffusion measures, calculated from the observed dataset and a suitable prior and likelihood function [7, 8]. Such an approach has been applied to brain DTI previously [8]. The posterior distribution represents epistemic uncertainty after using only the observed dataset to update a prior distribution,^1^ whereas the sampling distribution (even if estimated from a single available sample) represents uncertainty arising from the data generating process.

In the case of using bootstrapping to assess a smaller design by decimating a larger one, it is obvious that each sample should obey the design of interest. But in the case of UQ for diffusion measures obtained from fitting a single entire dataset, it is still the case that each bootstrap sample should have the same size and design as the observed dataset, that is, it should contain the same number of images for each diffusion weighting [9]. In bootstrapping terminology, the diffusion weightings are strata, that is, separate subgroups of the dataset. The fundamental difficulty for cDTI is that the error in images is highly non‐ideal (not following a well‐defined theoretical distribution) even in single myocardial voxels, and is generally spatially correlated across multiple voxels (and to some degree also over time, that is, over consecutive images). These correlated errors can also be called physiological noise and arise from uncompensated motion due to respiration, variations in cardiac phase, and even from image registration. This fundamental difficulty is intrinsically linked to the design goal of obtaining uncertainty in summary statistics over multiple myocardial voxels: in order for the latter quantity to be in any way meaningful, resampling must produce new datasets that respect these correlated errors.

Table 1 shows three categories of bootstrap method against three requirements for UQ of the sampling distribution given a single observed cDTI dataset. These requirements are the ability to handle “non‐ideal errors” and “spatial‐correlations”, and to perform “size‐matched” UQ (rather than performing UQ for a smaller dataset than the one available). In model‐based bootstrap, which includes wild bootstrap [10, 11] and residual bootstrap, fitting residuals for the observed dataset are resampled. Since the idealized assumptions about the error are unlikely to be valid representations of the true error distribution even within individual voxels [9], these methods are certainly not suitable for handling spatially correlated errors over multiple voxels.

**TABLE 1:** Bootstrap methods against three requirements for UQ in cDTI.

Bootstrap method	Non‐ideal errors	Spatial correlations	Size‐matched
Model‐based bootstrap			✓
Paired‐datasets bootstrap	✓	✓	
Repetition bootstrap	✓	✓	✓

The “paired‐datasets” bootstrap uses two (nominally) independent but equivalent datasets of size N to create new samples: for i=1…N, an image is chosen from either dataset A or B [6]. The two datasets can be considered as one dataset of size 2N with N strata each containing 2 images, where samples are generated by removing a random image from each strata (note that stratified bootstrapping is problematic for small strata [9], and here the strata have the minimum possible size). This method prevents duplicate images in any bootstrap sample, but it cannot provide UQ for diffusion measures derived from the full dataset actually available (size 2N), thus failing the size‐matched requirement.

This leaves only repetition bootstrap methods that use stratified bootstrapping, where resampling takes place within each strata (the set of repeated images with the same *b*‐value and *b*‐vector). For cDTI, whole images can be sampled with replacement (rather than handling each voxel independently) from each strata in order to account for spatial correlations. In particular, in “repetition bootknife” a random image is first “jackknifed” (removed) out of each strata before sampling with replacement from the remaining images [9]. Importantly, this method provides UQ for measures obtained from fitting the entire available dataset. This image‐level repetition bootknife method fulfills all of the criteria in Table 1, allowing UQ for voxel‐wise diffusion measures and summary statistics over myocardial voxels.

## 3 Methods

We apply UQ to a previously analyzed dataset of 11 healthy volunteers (HV) and 16 hypertrophic cardiomyopathy (HCM) patients: each dataset comprised b‐values of $100s/{\mathrm{mm}}^{2}$ (3 directions, 12 repetitions), and $450s/{\mathrm{mm}}^{2}$ (30 directions, 6 repetitions), for 3 short axis slices (apical, mid‐cavity, and basal), collected on a Siemens Prisma 3T MRI scanner (see [12] for details).

We refit these data with robust weighted least squares (RWLS) [13], and calculated diffusion measures MD, FA, ∣E2A∣, and HA. We then performed bootstrapping using image‐level repetition bootknife (restricted to myocardial voxels). We fit each sample with RWLS and recorded diffusion measure maps and summary statistics (average of diffusion measures in (i) each AHA segment; (ii) each slice; (iii) all voxels, i.e., global). We then calculated the standard deviation of diffusion measures per voxel (uncertainty maps) and the standard deviation of summary statistics.

Given the uncertainty maps, it is possible to calculate uncertainty weighted summary statistics. Here, we calculated inverse variance weighted averages (for (i–iii) as above), for both the original datasets and for each bootstrap sample. The latter allows to calculate the standard deviation of the uncertainty weighted summary statistics with respect to bootstrap samples. For angle‐based measures HA and E2A we used the circular standard deviation (using the “stats.circstd” function from SciPy [14]). For weighted averaging of ∣E2A∣, we utilized uncertainty in E2A rather than ∣E2A∣, to avoid assigning low and high angles a reduced uncertainty due to the magnitude operation.

Statistical comparison between HV and HCM was done via difference of group medians and the Mann–Whitney *U* test, for global averages (unweighted and weighted) of MD, FA, and ∣E2A∣.

Our code will be made available upon request.

## 4 Results

**FIGURE 1:**
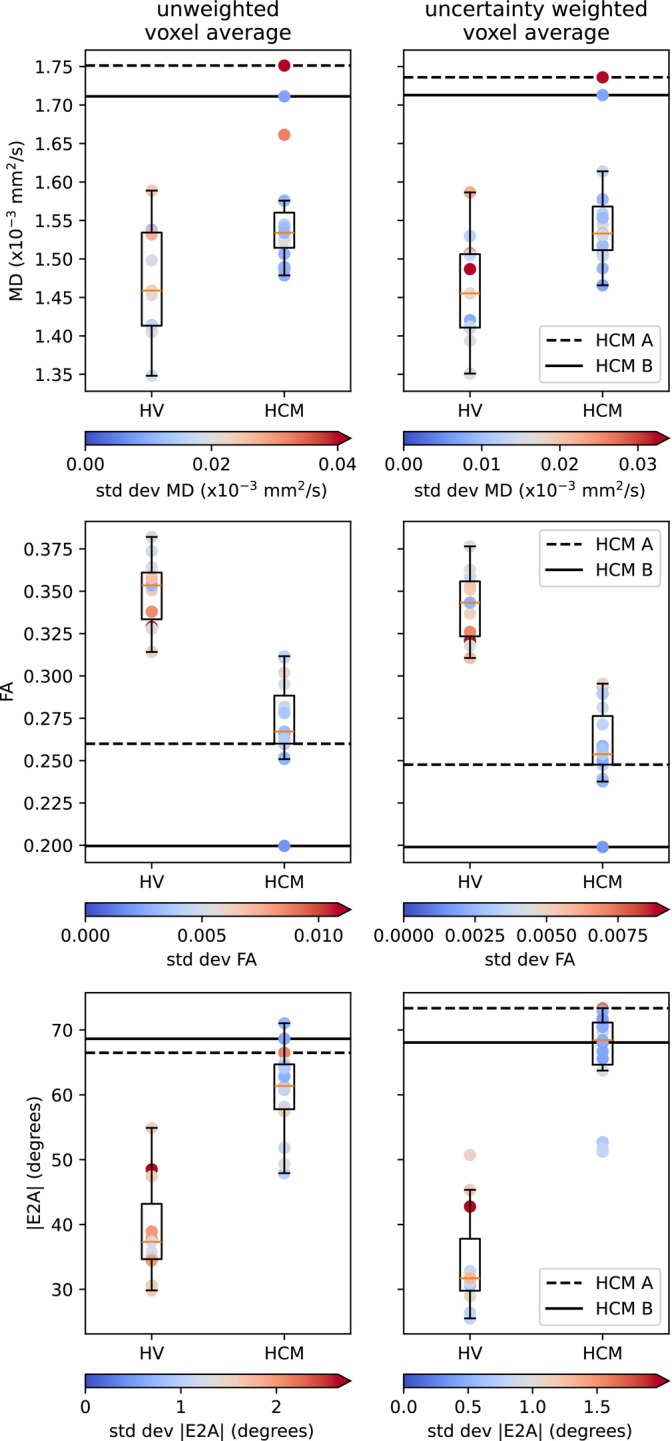
Diffusion measures (averages over myocardial voxels) for HV versus HCM patients, for unweighted and uncertainty weighted averages. The individual points representing subjects are colored by the standard deviation of the respective value.

Figure 1 shows global averages of diffusion measures for all subjects, for both unweighted and weighted averages. The individual points representing subjects are colored by the standard deviation of the respective value (the color range is 0 to 3 × 1.4826 × median absolute deviation of standard deviation over subjects, to aid visualization). Table 2 shows differences of group medians (orange lines in the boxplots of Figure 1) and *p* values. The group differences are larger and the *p* values smaller (or equal) for the weighted average. In case of ∣E2A∣, the group difference is $24.0^{{}^{\circ}}$ for unweighted averages and $36.7^{{}^{\circ}}$ for uncertainty weighted averages, an increase in difference of over 50%. Nearly all HV subjects see a decrease in global ∣E2A∣ with the weighted method, whereas nearly all HCM patients see an increase. The standard deviation of weighted averages is lower for all subjects, since the variance in AHA measures between bootstrap samples is reduced by inverse variance weighting.

**TABLE 2:** Difference of group medians of diffusion measures (HCM–HV) and *p* value (Mann–Whitney *U* test).

Methods	MD		FA		∣E2A∣	
	Diff	*p*	Diff	*p*	Diff	*p*
Unweighted average	0.075	0.022	−0.086	2.1e‐05	24.0	5.2e‐05
Uncertainty weighted average	0.078	0.0031	−0.090	2.1e‐05	36.7	2.1e‐05

**FIGURE 2:**
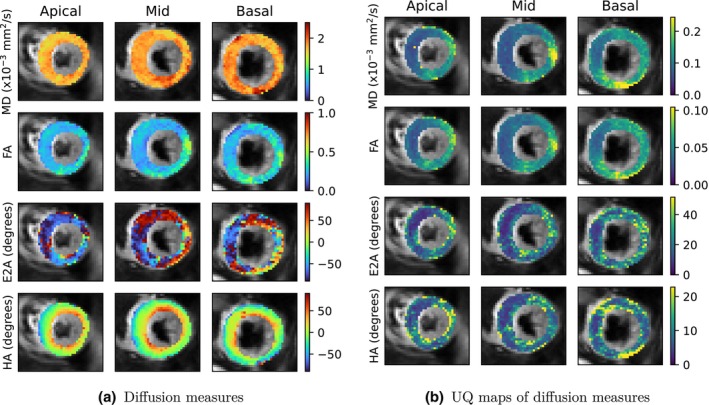
Diffusion and uncertainty quantification maps (standard deviation over bootstrap samples) for mean diffusivity (MD), fractional anisotropy (FA), secondary eigenvector angle (E2A), and helix angle (HA), for HCM A in Figure 1.

**FIGURE 3:**
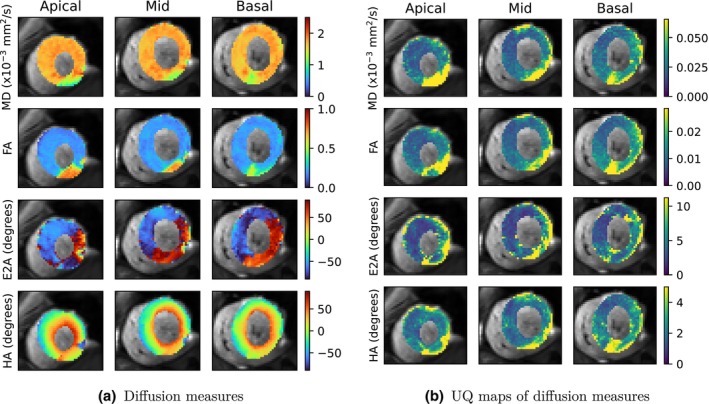
Diffusion and uncertainty quantification maps (standard deviation over bootstrap samples) for mean diffusivity (MD), fractional anisotropy (FA), secondary eigenvector angle (E2A), and helix angle (HA), for HCM B in Figure 1.

Figure 1 indicates two example HCM patients: diffusion measures and uncertainty maps for “HCM A” (Figure 2) and “HCM B” (Figure 3) are shown (in the Supporting Information we have included figures for two example cases of healthy volunteers). The scale for uncertainty maps is 0 to 3×1.4826×Median Absolute Deviation of voxel values. HCM A (Figure 2) has the highest MD and highest MD uncertainty of all subjects. Figure 2a shows high MD overall, but Figure 2b indicates high MD uncertainty with most values above $0.1\times 10^{-3}{\mathrm{mm}}^{2}/s$. The results for this subject should be treated with caution, despite the diffusion measures maps of Figure 2a not indicating a problematic scan (although a few small regions have very high MD, possibly from artifacts). HCM B (Figure 3) has the lowest FA and second highest MD. However, the uncertainty in both these values is low, and visual inspection of the diffusion maps in Figure 3a shows highly heterogeneous values except for clear artifacts that were excluded from the voxel summary statistics (notably, these artifacts also have very high uncertainty in Figure 3b). It is therefore reasonable to trust the diffusion measures for this subject despite appearing to be a group outlier for FA.

## 5 Discussion

We have demonstrated that a repetition bootstrap method can be used for uncertainty quantification of cDTI measures calculated by fitting the entire available dataset. This allows for both diffusion measure uncertainty maps and UQ of summary statistics over myocardial voxels. Furthermore, we have demonstrated potential benefits of calculating inverse variance weighted summary statistics. Results for a dataset of HV versus HCM patients suggest that group differences and significance levels are larger when using uncertainty weighted summary statistics. The overall higher ∣E2A∣ of the HCM group is increased by uncertainty weighted summary statistics, while the overall lower ∣E2A∣ of the HV group is decreased. Note that voxels with larger variances are more likely to contain larger errors, and uncertainty weighted summary statistics reduce the contribution from such voxels. However, future validation of uncertainty weighted summary statistics should be performed to better assess their application in CDTI. Note that it is further possible to calculate weighted medians over voxels, and to assess the co‐variance of different diffusion measures with respect to the sampling distribution. Although not shown here, the uncertainty for summary statistics can also be directly incorporated into group analysis.

In Section 2, we justified the use of repetition bootstrap, namely image‐level repetition bootknife, as being necessary to appropriately account for spatially correlated physiological noise in cDTI. Although there are likely unavoidable issues with repetition bootstrap methods generally, such as bias from having a finite sample or from having small strata (fewer repeats), it is still sensible to perform UQ in order to assess the reliability of diffusion measures. However, it is still important to assess the diffusion measures themselves, alongside the image dataset, to understand the presence of artifacts. We utilized a previously segmented dataset, where artifact masks had been defined without the use of UQ measures to identify and remove artifact regions from myocardial averages. While we found that these regions generally have high uncertainty (and as such, differences between unweighted and weighted averages over voxels were pre‐emptively reduced by exclusion of these regions), we cannot recommend that uncertainty measures should be used as a sole indicator of artifacts, nor that uncertainty weighting can fully compensate for the effects of artifacts on summary statistics.

Uncertainty quantification may pose a big dilemma for the design of acquisitions for cDTI: the larger the strata (i.e., the larger the number of repetitions) the more accurate the uncertainty quantification (estimation of the sampling distribution) is likely to be [9]. However, our work investigating the precision and bias of different cDTI dataset designs demonstrated that choosing more diffusion encoding directions with fewer repeats is preferable, given a total budget of images [5]. It is plausible to suppose that the most accurate diffusion measures are obtained with completely different directions and no repeats, in which case repetition bootknife is impossible. Note that the minimum size of strata for repetition bootknife is technically 2, but in this case the method reduces to jackknifing a random image from each strata (the remaining single image per strata will be sampled twice, but this does not change the result of fitting compared to sampling this remaining image only once). In this 2‐repetition case, one can see that the bootstrap samples are effectively equivalent to the “paired‐dataset” bootstrap. Nonetheless, this may be preferable to standard repetition bootstrap [9], and just further emphasizes the need to obtain enough repetitions.

This issue of requiring repetitions for UQ has implications for using deep‐learning to obtain cDTI maps with near‐minimal image acquisitions (e.g., 6 directions with 1 repeat and 1 $b_{0}$ image, as in [15]). Besides that such a small sample cannot really be representative of the data generating process even for breath‐hold acquisitions, it is impossible to bootstrap such datasets with repetition bootstrap since they do not contain repetitions. However, there is an opportunity here as well: if deep‐learning methods can focus on improving quality of cDTI measures from relatively few directions (previous work in the brain suggests 30 directions [16], also supported by our recent work for cDTI [5]), to better reflect results that would have been obtained with more directions, then it may be tractable to obtain image repeats with the time saved. This not only allows to perform UQ via repetition bootstrap and to have more accurate results (compared to the same number of directions with no repetitions), but would appropriately place a burden on deep‐learning methods to demonstrate their variability with respect to different samples of the data. But as already mentioned above, there is an irony in reducing directions in order to obtain repetitions for doing UQ, as compensating for reduced directions using deep‐learning is a more difficult task than reducing repetitions.

For the assessment of post‐processing pipelines and imaging parameters, it is possible that the bootstrapping method we have utilized here could be used to investigate sources of variance in diffusion measures. However, we would generally recommend that such assessments focus on accuracy and not only on variance, which requires a reference result in the form of a much larger dataset. Such work then falls into the category of using bootstrapping to assess the accuracy and precision of results from smaller datasets created by decimating a larger reference dataset. Nonetheless, the bootstrapping methods used here could still be used to assess how the variance of diffusion measures might change with respect to modifications of the post‐processing pipeline, but we have not explored that application in this technical note.

## 6 Conclusion

Uncertainty quantification in cDTI can be performed using repetition bootstrap methods where sampling is performed at the image level. This uncertainty quantification allows for estimation of the variance of the sampling distribution of diffusion measures, both on a voxel‐level and on a summary statistic level. Furthermore, it is then possible to calculate uncertainty weighted summary statistics over myocardial voxels. cDTI research, including that seeking to minimize the size of the acquisition, must ensure the acquisition design is suitable to enable estimation of the sampling distribution of resulting diffusion measures. Importantly, UQ may guide interpretation of diffusion parameter maps and facilitate clinical applicability of cDTI.

## Funding

This work was supported by Wellcome Trust Investigator (219536/Z/19/Z) and British Heart Foundation (PG/19/1/34076; PG/24/12118).

## Supporting information


**Figure S1:** Diffusion measures for all subjects.
**Figure S2:** Diffusion maps for HV A in Figure S1.
**Figure S3:** Diffusion maps for HV B in Figure S1.

## Data Availability

Research data are not shared.

## ENDNOTES

^1^
The well‐known simplifying priors for linear models utilized in [8] result in less than 1.25% of tensors sampled from the prior being positive semi‐definite, which suggests the prior is misspecified.
