# Supplementary material for: Uncertainty Quantification for Cardiac Diffusion Tensor Imaging Without Additional Datasets
Source: Magn Reson Med. 2026 May 10;96(3):1458–63. doi: 10.1002/mrm.70414 (PMC13327503; doi:10.1002/mrm.70414)
Supplement: Supplementary file 1 — Figure S1: Diffusion measures for all subjects. Figure S2: Diffusion maps for HV A in Figure S1. Figure S3: Diffusion maps for HV B in Figure S1. [file MRM-96-1458-s001.pdf]

Supplementary file for "Uncertainty Quantification for Cardiac Diffusion Tensor Imaging without additional datasets"

Sam Coveney, Irvin Teh,
May Lwin, Mehak Asad, Isaac Watson, Maryam Afzali,
Erica Dall'Armellina, Jurgen E Schneider

January 2026

Figure S1 is a duplicate of Figure 1 in the main manuscript, except highlighting two healthy volunteers: HV A (Figure S2) has the highest uncertainty for MD and HV B (Figure S3) has the lowest uncertainty for MD. Note that in both cases, regions of artifact visible in the DTI measures and raw images (identified in a previous analysis) were excluded from averages over voxels. It is worth noting that corrupted DTI measures have larger uncertainties, which is particularly clear for Figure S3.

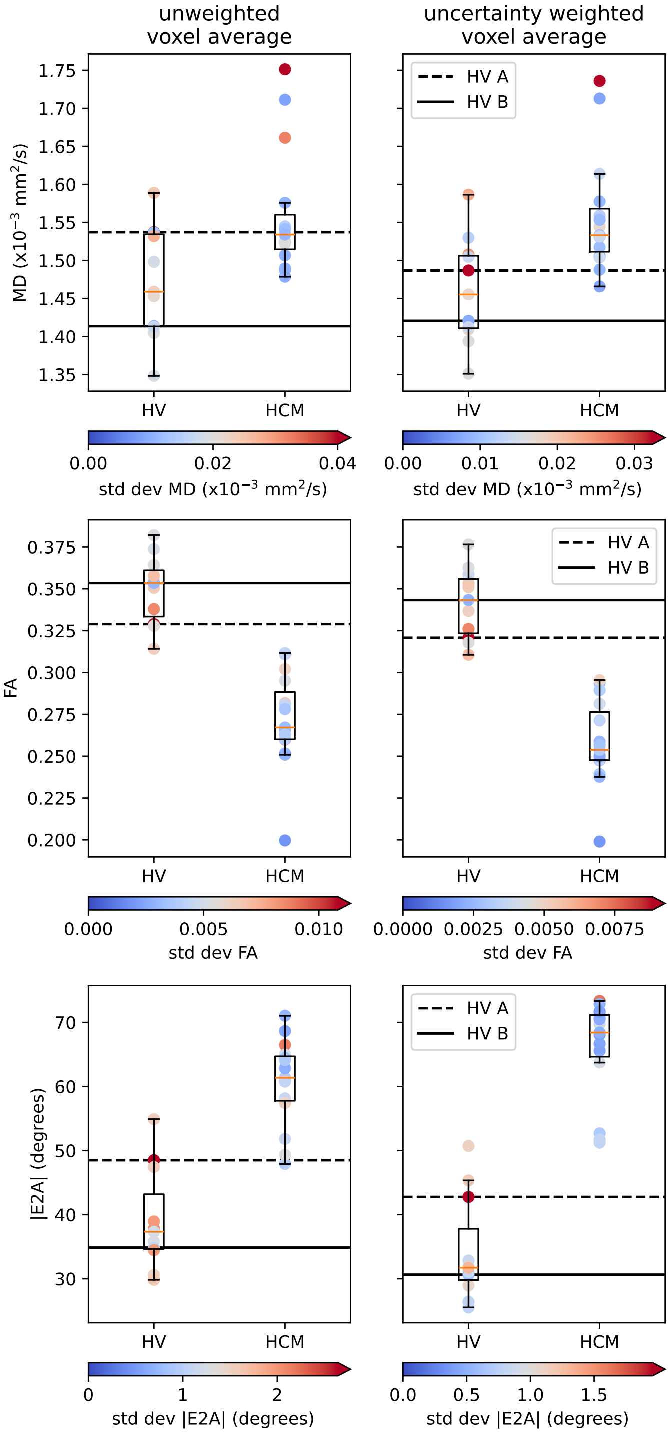

Figure S1: Diffusion measures for all subjects.

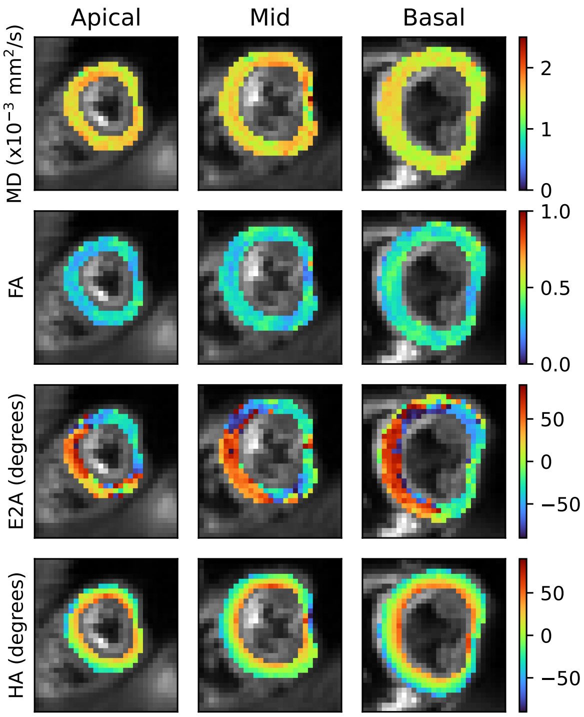

(a) Diffusion measures

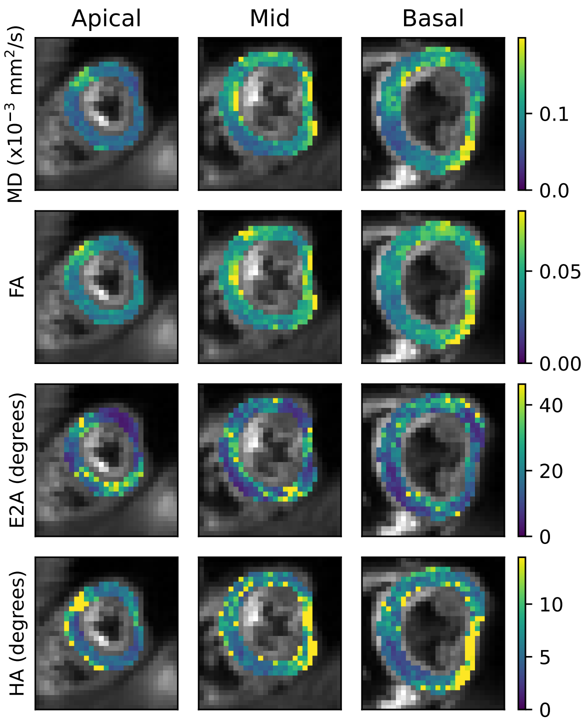

(b) UQ maps of diffusion measures

Figure S2: Diffusion maps for HV A in Figure S1.

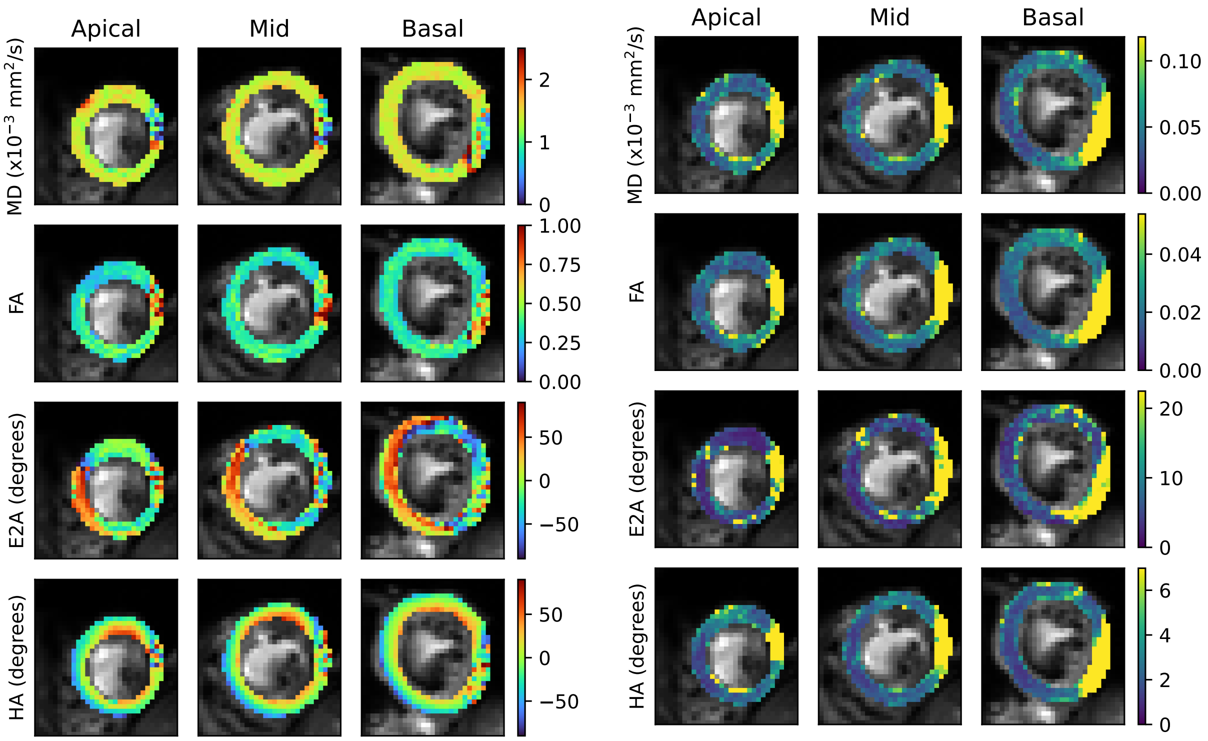

(a) Diffusion measures **(b)** UQ maps of diffusion measures

Figure S3: Diffusion maps for HV B in Figure S1.
